# Neuroprotective effects of some epigenetic modifying drugs’ on *Chlamydia pneumoniae*-induced neuroinflammation: A novel model

**DOI:** 10.1371/journal.pone.0260633

**Published:** 2021-11-30

**Authors:** Elif Kaya-Tilki, Miriş Dikmen

**Affiliations:** Department of Pharmacology, Faculty of Pharmacy, Anadolu University, Eskisehir, Turkey; National Institute of Allergy and Infectious Diseases, UNITED STATES

## Abstract

*Chlamydia pneumoniae (Cpn)* is a gram-negative intracellular pathogen that causes a variety of pulmonary diseases, and there is growing evidence that it may play a role in Alzheimer’s disease (AD) pathogenesis. Cpn can interact functionally with host histones, altering the host’s epigenetic regulatory system by introducing bacterial products into the host tissue and inducing a persistent inflammatory response. Because Cpn is difficult to propagate, isolate, and detect, a modified LPS-like neuroinflammation model was established using lyophilized cell free supernatant (CFS) obtained from infected cell cultures, and the effects of CFS were compared to LPS. The neuroprotective effects of Trichostatin A (TSA), givinostat, and RG108, which are effective on epigenetic mechanisms, and the antibiotic rifampin, were studied in this newly introduced model and in the presence of amyloid beta (Aβ) 1–42. The neuroprotective effects of the drugs, as well as the effects of CFS and LPS, were evaluated in Aβ-induced neurotoxicity using a real-time cell analysis system, total ROS, and apoptotic impact. TSA, RG108, givinostat, and rifampin all demonstrated neuroprotective effects in both this novel model and Aβ-induced neurotoxicity. The findings are expected to provide early evidence on neuroprotective actions against Cpn-induced neuroinflammation and Aβ-induced neurotoxicity, which could represent a new treatment option for AD, for which there are currently few treatment options.

## 1. Introduction

Alzheimer’s disease (AD) is a chronic multifactorial neurodegenerative disease that accounts for 75–80% of dementia [1]. The initial detectable disorder is the accumulation of 42 amino acid-long Aβ protein in extracellular plaques in the brain decades before clinical onset. AD is a slow-progressing disease with no cure [2]. Neurodegeneration ultimately leads to the AD clinical syndrome, which is associated with Aβ aggregation accompanied by synaptic disruption and increased tau phosphorylation and secretion. The AD clinical state is defined by neurological symptoms that worsen as the disease advances. This is the most common pathophysiological explanation for AD [3]. The cellular pathways causing these anomalies are still being investigated, but oxidative stress [4], energy deprivation [5, 6], and neuroinflammation [7, 8] are thought to be critical processes that initiate and/or exacerbate the pathophysiological substrates of AD [9], which were originally defined as extracellular cortical plaques containing Aβ, and intraneuronal tangles containing aggregated tau protein [10]. However, practically all clinical trials that have targeted these substrates have failed to identify any effective treatments for AD. As a result of this, scientists are gradually shifting away from the basic assumption that Aβ caused the disease to different hypotheses of pathogenesis [11].

Neuroinflammation is a response involving all existing cells in the central nervous system, including neurons, macroglia and microglia. The activation of microglia is the first sign of neuroinflammation. They are found around senile plaques when they are triggered by a variety of factors including trauma, age, genetic history, environmental factors and epigenetics, stress or protein aggregates such as Aβ fibrils and tau double helix filaments [12, 13]. Recently, it has been debated whether neuroinflammation is the key driver of neurodegeneration and/or downstream consequences of Aβ [14]. The “pathogen hypothesis,” also known as the “infection hypothesis” in AD, proposes that chronic infection by virus, bacterial, and/or fungal infections could be a cause for sporadic AD onset during aging [15]. Activation of the immune system and long-term inflammation caused by chronic microbial infections allow microbial pathogens and/or their products to pass across the blood–brain barrier [16]. They associate with Aβ in the brain and produce Aβ fibrillation, which results in the formation of senile plaques [17]. Gliosis is exacerbated by the disruption of the blood–brain barrier, which permits peripheral inflammatory chemicals and cells to enter the brain and induce gliosis [18]. Additionally, a multitude of risk factors, including heredity, stress, sleep, diet, traumatic brain injury, and aging, all influence the progression of the condition. The combination of these elements results in a vicious inflammatory response that is aggravated by chronic infections or reactivation, and which ultimately results in chronic neuroinflammation [19]. The eventual effect of this process is neuron loss and the development of AD pathology [8]. “Antimicrobial protection hypothesis” suggested in this context is that the antimicrobial functions of Aβ plaques and tau tangles, which are hallmarks of AD, develop into neuroprotective responses that protect against neuroinflammation, leading to later neurotoxic effects [15]. According to this theory, amyloid deposition, is an early innate immune response to a genuine or perceived immunothreat. Aβ captures and neutralizes pathogens that are invading. To fight infection and eliminate amyloid/pathogen deposits, it triggers neuroinflammatory pathways. Neurodegeneration and inflammation are caused by chronic activation of this system in AD [20]. While preserving the same molecular suspect, this idea proposes an altogether new strategy to AD that does not include eliminating or inhibiting Aβ or its cleavage. Instead, an alternate method would be to locate probable favored sources of infection [21].

Several pathogens have been identified as candidates for the "pathogen hypothesis" over time, including *Chlamydia pneumoniae* (Cpn), an obligate intracellular bacterium and respiratory tract pathogen that infects mucosal surfaces, especially the lung/pulmonary and nasal mucosa [22]. The SET domain protein (cpnSET) from this bacteria was the first to be discovered methylating the chlamydial histone-like proteins HC1/HC2 and it is suggested that Cpn functionally interacts with host histones [23]. The epigenetic regulatory system of host cells can be altered by pathogenic bacteria by introducing bacterial products into the host tissue and evoking a persistent inflammatory response [24]. It has been demonstrated that, regardless of genetic code defects, epigenetic inconsistency influences the development of AD [25–29]. In this context, considering pathogens associated with AD pathogenesis, including Cpn, as epigenetic modifiers may be promising for treatment because epigenetic modifications can be reversed whereas genetic mutations cannot. The epigenetic landscape of a cell is determined by DNA methylation, histone variations and modifications, and nucleosome placement [30]. By modifying chromatin structure, acetylating promoter areas, or deactivating co-repressors, histone deacetylase inhibitors (HDACi) can modify gene expression [31]. DNA methyltransferase inhibitors (DNMTi) are genome hypomethylating agents that promote the reactivation of silenced genes by DNA methylation [32]. Despite the fact that epigenetic modifiers are used to treat cancer, variations in gene methylation have been linked to neurological disorders [33, 34], aging, and neurodegenerative diseases [35]. Since epigenetic machinery components have been effectively employed in preclinical studies for numerous diseases, including neurodegenerative diseases [36], an epigenetic-based approach for treating these disorders seems promising.

The aim of this research was to optimize a novel Cpn-induced *in vitro* neuroinflammation model. There are a number of neuroprotective effect studies in the literature that use neuron models co-cultured with microglia activated by a commercially available, commonly lyophilized, inductive agent such as LPS or Aβ [37–40]. *In vitro* Cpn studies, on the other hand, have typically studied infected cells that are unable to distinguish between live and dead bacteria, and the analysis cannot be confined to living cells [41–43]. Therefore, a more convenient neuroinflammation model for molecular research in pharmacology was introduced in this study, to carry out a related neuroinflammation model with lyophilized Cpn lysate and to maintain accuracy without returning to the inoculation stage before each experiment. Following model optimization, the neuroprotective effects of histone deacetylase inhibitors trichostatin A (TSA) and givinostat, a DNA methyltransferase inhibitor RG108, and rifampin, an antibiotic used to treat this infection that has previously been shown to have neuroprotective effects [44–46], were investigated in this model and in the presence of Cpn, LPS and Aβ.

## 2. Methods

### 2.1. Chemicals

All chemicals and reagents used in this study were of analytical grade and used as received without any further purification. RPMI-1640, Eagle’s Minimum Essential Medium (EMEM) medium, fetal bovine serum (FBS), phosphate buffered saline (PBS), dimethyl sulfoxide (DMSO), phorbol 12-myristate 13-acetate (PMA), lipopolysaccharide (LPS), interferon-γ (IFN-γ), retinoic acid (RA) were purchased from Sigma Aldrich (St. Louis, MO, USA). RG108, givinostat (ITF2357), trichostatin A and rifampicin were purchased from Selleckchem (Germany).

### 2.2. Cell culture and treatments

SH-SY5Y human neuroblastoma (ATCC CRL-2266), HEp-2 human epithelial carcinoma cell line (ATCC CCL-23), and HMC3 human microglia cells (ATCC CRL-3304) were cultured in Eagle’s Minimum Essential Medium (EMEM), THP-1 human monocyte cells (ATCC TIB-202) were cultured in RPMI-1640 media containing 10% fetal bovine serum and 1% penicillin/streptomycin. To reach 70–80% density, the cells were cultured in a Herceles™ CO2 incubator (Thermo Scientific, USA) at 37°C with 5% CO2.To differentiate the SH-SY5Y cells into neuronal fenotype, the current medium was changed with 1% FBS, 1% penicillin/streptomycin, and 10 μM RA, which was then incubated for 5 days [47]. 24-h incubation in 100 ng/ml PMA containing serum-free medium, monocytes were polarized into M0 macrophages and rested for 48-h in growth medium [48]. DMSO-dissolved givinostat (ITF2357), trichostatin A, and rifampicin were diluted with fresh medium to desired concentrations. The control group was prepared with 0.1% DMSO in medium in experiments involving drugs.

### 2.3. Development of a *Chlamydia pneumonia*e-induced neuroinflammation cell model using lyophilized Cell-Free Supernatant (CFS)

#### 2.3.1. *Chlamydia pneumoniae* culture in HEp-2 cells and preparation of lysates

HEp-2 cells were used as a host to inoculate Cpn. A 6-well plate of 1X10^6^ HEp-2 cells was seeded 48 hours prior to inoculation with Cpn (ATCC 53592) as previously described [49]. Briefly, the suspension of elementary bodies diluted in infection medium was added directly to wells and the mixture was centrifuged at 1500 × g for 1 h followed by 1 h incubation at 37°C in the presence of 5% CO_2_. Current medium was discarded and cells were washed with 300 μL Hanks Balanced Salt Solution and 500 μL fresh medium was added to the wells. After 72 hours, the inclusion bodies were confirmed using the Pathfinder Chlamydia Culture Confirmation System (Cat. No. 30701, Bio-Rad, Germany) and the cells were imaged using the Cytation 3 Cell Imaging Multi-Mode Reader (BioTek, USA). The number of inclusion-forming units per milliliter (IFU/ml) in HEp-2 cells was used to determine the infectivity titers of chlamydial stocks and 1x10^6^ HEp-2 monolayers in 6-well plate were contaminated with Cpn suspended in inoculating media at 1 multiplicity of infection (MOI) ratio. In order to find the Cpn lysate which provides microglia activation most similarly to LPS, various lysates were obtained, lyophilized and stored in aliquots at -80°C in the following ways: CFS collected from the wells (doi.org/10.17504/protocols.io.bzw2p7ge), lysate of the cells in supernatant homogenized using MagNA Lyser Green Beads in the MagNA Lyser Instrument (Roche Diagnostics, Germany), scratched cells in supernatant UV-inactivated for 40 minutes by exposing to UV irradiation at a distance of 15 cm from a UV germicidal light source (not homogenized) and scratched cells in supernatant, UV-inactivated and homogenized.

#### 2.3.2. Screening of lyophilized *Chlamydia pneumoniae* samples on proinflammatory cytokine levels

To compare the efficacy of lysates, levels of proinflammatory CD218a (IL-18R1) and IL-1β cytokines were measured in M0 macrophage-polarized THP-1 cells, by flow cytometry. 5x10^5^ M0 macrophages were treated with lyophilized samples diluted at 1 MOI in 24-well plates. Samples were collected and centrifuged at 110 rpm, then washing and incubation procedures were carried out in accordance with the CD218a (IL-18R) (Cat. No. 564675, BD Pharmingen, USA) and IL-1β (Cat. No. 508206, BioLegend, USA) kit procedures after 24-h. Antibody levels were determined using a BD Accuri C6 Flow Cytometer, and the data was analyzed using the same device’s analysis software (BD Accuri C6 Software).

#### 2.3.3. Determination of CFS concentration that activates microglia cells

LPS and/or proinflammatory cytokines like IFN-ɣ, have been used alone or in combination in most *in vitro* neuroinflammation models to study microglial activation [50]. Therefore, the effects of lyophilized cell free supernatant (CFS)+IFN-ɣ combination, which was determined to increase IL-1β and CD218a cytokine levels most effectively, on IL-1β, TNF-α and IL-8 gene expressions were compared with LPS+IFN-ɣ combination in order to determine the activation concentration.

1x10^6^ HMC3 cells were seeded on 6-well plates and treated with CFS or LPS alone or in combination with IFN-ɣ (10 ng/ml) at 1, 10 and 100 μg/ml concentrations. RNA isolations were performed in MagNA Pure Compact System (Roche, Germany) using MagNA Pure Compact RNA Isolation Kit® (Catalog No: 04802993001, Roche, Germany) after 24-h. The total RNA amounts of the samples were measured at 260 and 280 nm in the NanoDrop 2000® (Thermo Fisher, USA) spectrophotometer. cDNA was obtained using the Transcriptor High Fidelity cDNA Synthesis Kit® (Catalog no: 05091284001, Roche, Germany) following the kit protocol from 500 ng/μl RNA of each sample. After the cDNA samples were replicated by PCR method, the expression levels were determined with the LightCycler® 480 Real-Time PCR System (Roche, Germany) using the IL-1β, TNF-α and IL-8 primers (PRZ BioTech, Turkey). Actin beta (ACTB) was used as a housekeeping gene. Primer sequences and RT-PCR cycling conditions are shown in S1 and S2 Tables. Results were analyzed according to the changes in the amplification levels compared to the control group with the analysis software of the instrument.

#### 2.3.4. Determination of active microglia marker CD11b induction with CFS

To evaluate the concentration of CFS for microglia activation in the co-culture model, the effects of 10, 100 ng/ml and 1 μg/ml CFS concentrations with IFN-ɣ on the active microglia marker CD11b were compared with the most effective concentration of LPS (100 ng / ml)+IFN-ɣ for 24 hours. HMC3 microglia cells were seeded in 12-well plates at a density of 75x10^3^ cells per well and treated with treatment groups for 24 h. Then, cells were detached and centrifuged with Cell Staining Buffer (BioLegend, USA, Cat. No: 420201) for 5 minutes and the supernatant was discarded. Blocking was performed with 5 μl Human TruStain Fcx (BioLegend, USA, Cat. No: 422302) Fc receptor blocker solution diluted in 100 μl Cell Staining Buffer for 10 minutes at room temperature to reduce non-specific binding. The blocker was removed by centrifugation and the cells were stained with 5 μl PE anti-mouse/human CD11b Antibody (BioLegend, USA, Cat. No: 101208) diluted 1:5 in 20 μl Cell Staining Buffer for 15 minutes on ice in the dark. Then, the cells were washed 2 times with 2 ml Cell Staining Buffer and analyzed within 500 μl buffer using the BD Accuri ™ C6 flow cytometer.

#### 2.3.5. Monitoring cell viability of *Chlamydia pneumoniae*-induced neuroinflammation co-culture model with Real-Time Cell Analysis System

The xCELLigence Real-Time Cell Analysis System is a platform that displays electrical impedance as unitless cell index data (CI) on plates with interlocking gold microelectrodes to non-invasively track cell viability in real-time [51]. The E-plate Inserts inserted in special plates containing gold electrodes used in this device allow for real-time monitoring of cell-cell interactions with co-culturing [52]. CI values are used as an adhesion metric. When no cells are present, the CI value is zero, and it rises as cells adhere to the plate. The net cellular adhesion within the well is measured by xCELLigence [53].

To monitor the model’s cell viability in real time, SH-SY5Y cells were seeded at 5x10^3^ cells per well on E-plates, and incubated for 5 days within the instrument to induce differentiation [54]. 48 hours before the co-culture model was applied by inserting E-plate Inserts inside the E-plates, HMC3 cells were plated in EMEM medium containing 10% FBS and 1% penicillin/streptomycin onto E-plate Inserts at a density of 5x10^3^ cells per well. After 24 h, the existing medium was replaced with CFS (1 μg/ml) + IFN-ɣ, LPS (100 ng/ml) + IFN-ɣ or only growth medium (as control) containing fresh media for microglia activation and incubated for another 24 h. After activation, the medium was discarded and the cells were washed with PBS. E-plate Inserts were mounted on E-plates containing differentiated SH-SY5Y cells, which were previously washed in the same manner, and differentiation medium were added to the wells. In addition to co-culture groups, differentiated SH-SY5Y cells were also treated with the same groups without the E-plate Inserts. Cell viability were recorded in real time during the experiment.

### 2.4. Determination of drug concentrations with Real-Time Cell Analysis System

Cell proliferation analysis was performed with xCELLigence Real-Time Cell Analysis System to assess TSA, RG108, givinostat, and rifampin concentrations for use in both models. Differentiated SH-SY5Y and HMC3 microglia cells were seeded at a 5x10^3^ density per well and incubated 24 h until CI values reached a log phase. The system stopped at the end of 24 h, 100, 10 and 1 μM TSA, givinostat, RG108 and rifampin were applied on current media and the cell viability was tracked. The half maximal inhibitory concentration (IC_50_) values of 24th and 48th hours were evaluated in analysis software of the instrument. Simultaneously, slope plots were drawn using the CI values at 24 and 48 hours, and the statistical significance of the values discovered by assuming the average CI values of the control wells to be 100% was calculated.

### 2.5. Evaluation of epigenetic modifying drugs’ neuroprotective effects against Aβ-induced neurotoxicity

#### 2.5.1. Cell viability analysis against Aβ-induced neurotoxicity with Real-Time Cell Analysis System

Pathological deterioration in AD is mainly associated with overproduction of the Aβ peptide and the Aβ (1–42) variant is the toxic form that is more predominant in Alzheimer’s patients [55]. In this study, a modified oligomeric Aβ (1–42) neurotoxicity analysis protocol was applied in order to elucidate the effects of treatment groups on SH-SY5Y cell viability in the presence of Aβ [56]. In order to model Aβ-induced neurotoxicity *in vitro*, an oligomeric form of the Aβ1–42 peptide was prepared by diluting with ice-cold Ham’s F12 Nutrient Mixture medium dissolved in PBS containing 0.02 M NaOH at a final concentration of 50 μM and kept in a 37°C incubator for 7 days to form peptide aggregates prior to the experiments [57, 58]. 5x10^3^ SH-SY5Y cells were differentiated inside the xCELLigence instrument for 5 days and treated with 100 nM TSA, RG108, givinostat, rifampin; 100 ng/ml LPS+IFN-ɣ or 1 μg/ml CFS+IFN-ɣ for 24 h in order to evaluate neuroprotection. The co-culture was performed in the same way as the neuroinflammation model. Neurotoxicity was obtained instead of neuroinflammation by diluting to 5 μM with fresh medium and adding Aβ1–42 aggregates to the wells for 24 h in 100 μl of medium. Cell viability data was then monitored for 24 h and analyzed within the instrument.

#### 2.5.2. Determination of apoptotic effect with Annexin V-PI staining

To determine neuronal apoptosis associated with Aβ exposure, 25x10^3^ differentiated SH-SY5Y cells were seeded in 24-well plates and Aβ was applied. After 24 h, cells were trypsinized and staining was performed according to the CF 488A Annexin V and PI Apoptosis Kit (Biotium, USA, Cat. No: 30061) protocol. After washing the cells with 1xPBS, the working solution was prepared by diluting the PI with Annexin V binding buffer included in the kit. 5 μl Annexin V and 2 μl PI working solution were added to the centrifuge tubes and binded with 100 μl Annexin V binding buffer. After the samples were incubated for 30 minutes on ice and in the dark, 400 μl of Annexin V binding buffer was added to each tube and the samples were measured and analyzed in a BD Accuri™ C6 flow cytometry device [59].

#### 2.5.3. Determination of reactive oxygen species

The Total ROS/Superoxide Detection Kit (ENZ-51010, Enzo) was used according to the kit process for evaluating the protective effects of the treatment groups on SH-SY5Y cells seeded at a 2x10^4^ density per well in 96-well plates and Aβ was applied. ROS inhibitor N-acetyl cysteine (NAC) was applied at 5 mM concentration to negative control groups 30 minutes before the experiment and after incubation, 100 μL of “ROS/Superoxide Detection Solution” was added to the wells in the kit. The same procedure was done without the application of ROS inhibitor. Fluorometric analysis of stained cells was analyzed with Cytation 3 Cell Imaging Multi-Mode Reader (BioTek) using 490/525 nm and 550/620 nm filter sets [60].

### 2.6. Statistical analysis

The data generated in each experiment was imported into the GraphPad Prism 7.0 software and replicates were averaged and the standard deviations were calculated. Graphics were drawn on the same software and the data was statistically analyzed with one-way analysis of variance (ANOVA) and Tukey’s post hoc test. The results are expressed as the means of three independent experiments (n = 8 for cell viability assays, n = 3 for others) ± standard deviation (SD) and p <0.05 *, p <0.01 **, p <0.001 ***, p <0.0001 **** were considered to be significant compared to the control group. p> 0.05 values were accepted as non-significant.

## 3. Results

### 3.1. Optimization of *Chlamydia pneumonia*e-induced neuroinflammation cell model

Since Cpn is an organism difficult to isolate in cell culture, the HEp-2 cell line, which is a human epithelial carcinoma cell, was used as a host and Cpn inoculation was provided. Unlike other cell lines, the HEp-2 cell line was selected for Cpn culture because it does not need passage or pretreatment, and its properties are well known and can be obtained commercially [61]. The inoculation formation was confirmed by genus specific antibody dye (S1 Fig).

#### 3.1.1. Effects of lyophilized *Chlamydia pneumoniae* samples on proinflammatory cytokine levels

**Fig 1:**
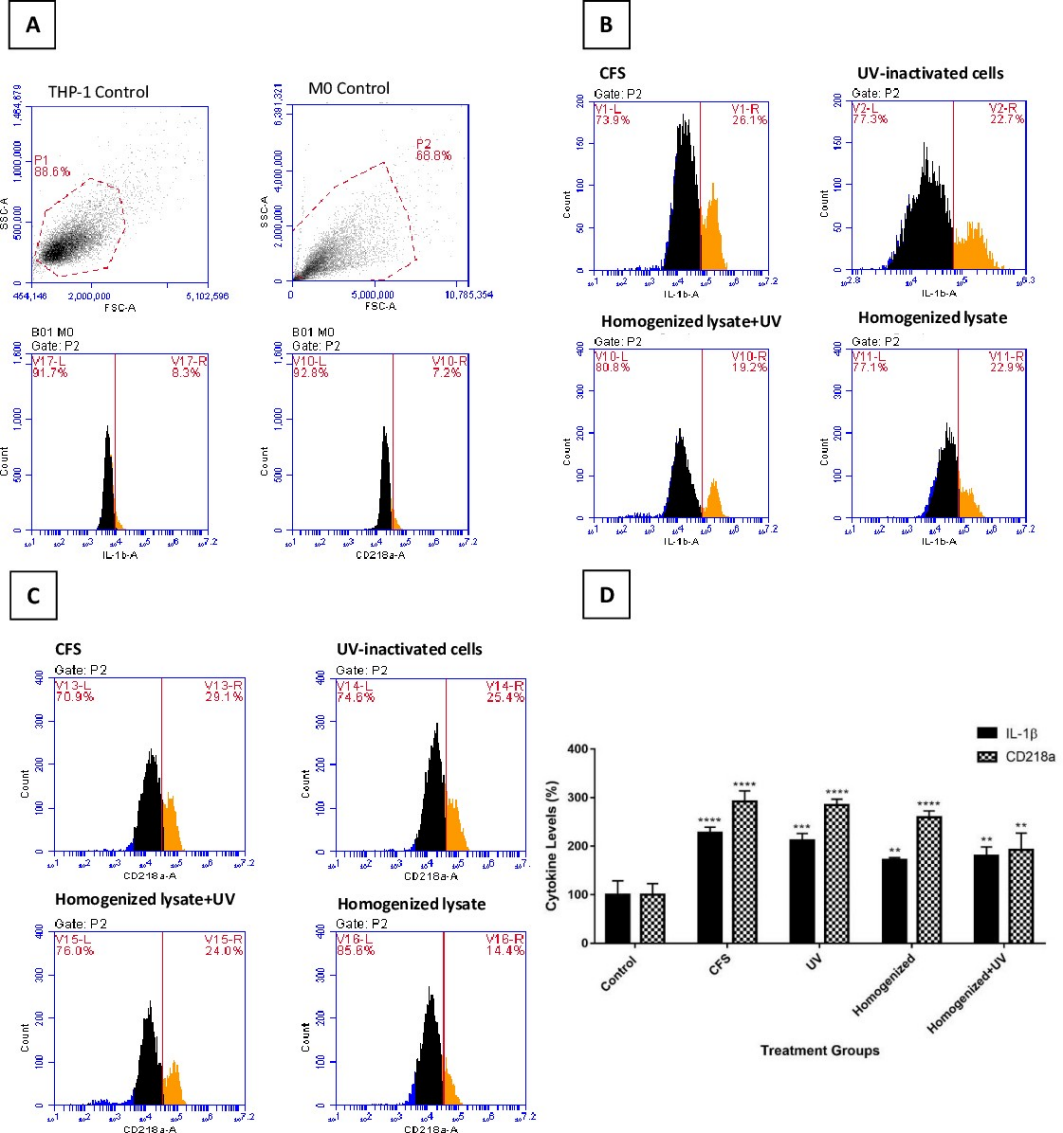
IL-1β and CD218a cytokine levels in THP-1 cells that have differentiated into M0 macrophages and have been stimulated with various Cpn lysates. (A) THP-1 control shows monocytes, M0 control shows gated scatter. Basal IL-1β and CD218a were also measured in unstimulated M0 macrophages. (B) IL-1β and (C) CD218a levels in M0 macrophages treated with CFS, non-homogenized UV-inactivated cells, homogenized lysate, or UV-inactivated homogenized lysate. One representative result from three independent repeats is shown. (D) The bar graph shows the % change in cytokine levels in the treatment groups. The IL-1β and CD218a levels of untreated M0 macrophages were used as a control group and the changes in cytokine levels of treatment groups were calculated according to these groups. The data represents the means ± SDs of three individual experiments (n = 3 for each). One-way ANOVA was used for statistical analysis for each variant, followed by a post hoc Tukey’s multiple comparisons test. no difference: p> 0.05; significant difference: * p < 0.05, ** p < 0.01, *** p < 0.001, **** p < 0.0001 vs. untreated controls.

As differentiated THP-1 cells are generally used as a model in neuroinflammation studies [62–65], the efficiency of Cpn samples on proinflammatory cytokines activated by different pathways was compared in the cytokine study. According to the results of pro-inflammatory cytokine measurement, untreated M0 macrophages, CFS, homogenized lysate of cells in supernatant, UV-inactivated scratched cells with or without homogenization were increased IL-1β levels by 8.3, 26.1, 22.9, 19.2 and 22.7%; and CD218a levels by 7.2, 29.1, 14.4, 24 and 25.4%, respectively (Fig 1). Based on the results of the experiment, CFS was chosen as the microglia inducer in the neuroinflammation model since it is easier to obtain and elevates cytokine levels higher than other lysates.

#### 3.1.2. Effects of CFS concentrations on IL-1β, TNF-α and IL-8 gene expression levels of microglia cells

In order to evaluate the CFS concentrations that activate HMC3 microglia cells, the effects of the different CFS and LPS concentrations on proinflammatory cytokine activation using RT-PCR were compared. Many studies have characterized glial activation after treatment with LPS or IFN-ɣ and tested the potential anti-inflammatory effects of candidate compounds. Since IFN- ɣ stimulates the immune response in cells, LPS and IFN-ɣ commonly use in combinations in inflammatory models and enhances inductive effects of LPS. In particular, high levels of IFN- ɣ have been reported in various neurological disorders and animal models, including AD [66].

**Fig 2:**
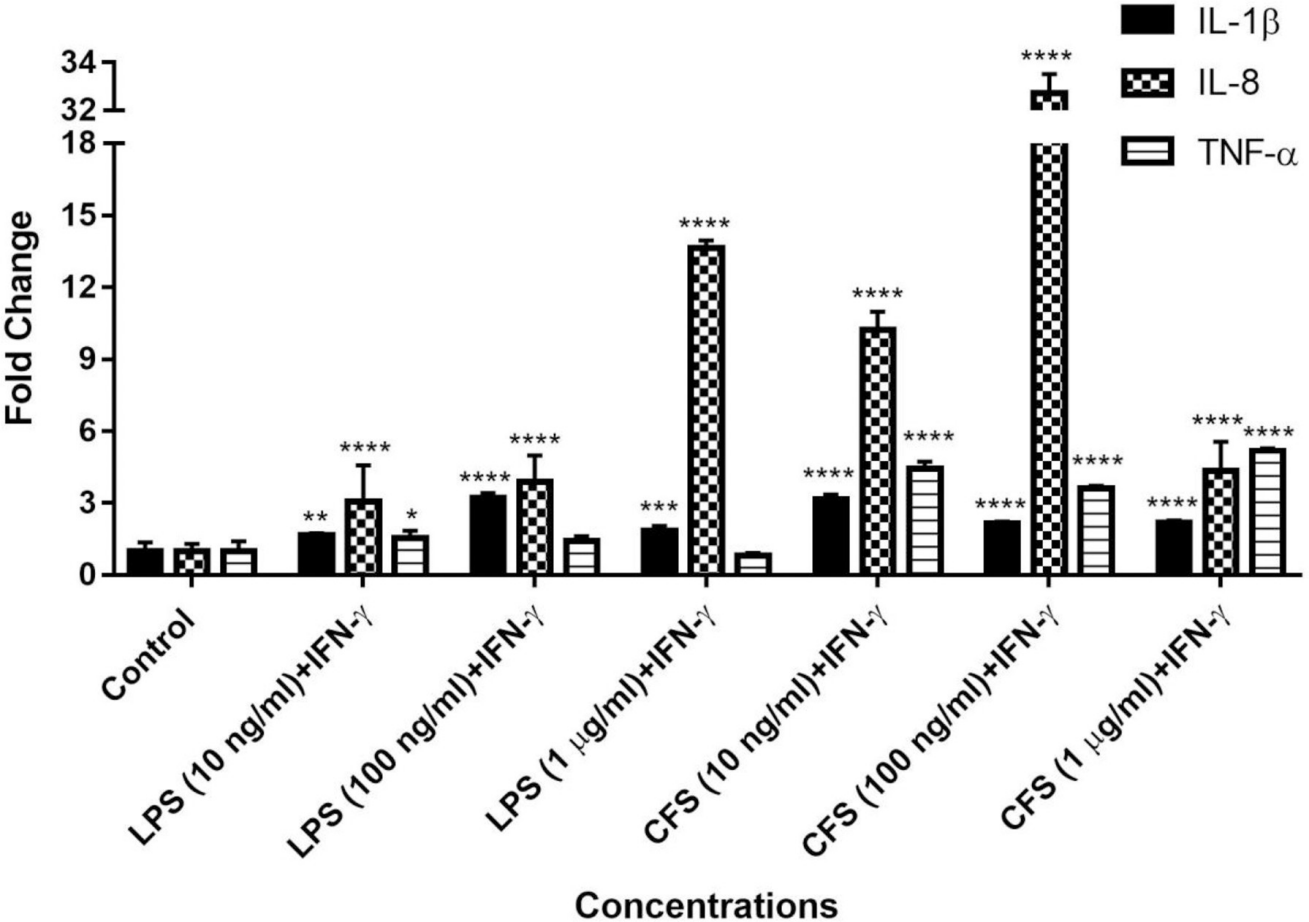
RT-PCR analysis for gene expression levels of IL-1β, TNF-α and IL-8 of HMC3 cells. The data represents the means ± SDs of three individual experiments (n = 3 for each). One-way ANOVA was used for statistical analysis for each group, followed by a post hoc Tukey’s multiple comparisons test. All of the results are displayed on a single graph. no difference: p> 0.05; significant difference: *p < 0.05, **p < 0.01, *** p < 0.001, **** p < 0.0001 vs. untreated controls.

According to the results, IL-1β expression levels were increased with 10, 100 ng / ml and 1 μg / ml LPS or CFS in combination with 10 ng / ml IFN-ɣ 1.64, 3.21, 1.83 and 3.16, 2.14, 2.17 fold for IL-1β; 1.55, 1.41, 0.82 and 4.42, 3.62, 5.18 for TNF-α; 3.07, 3.88, 13.64 and 10.23, 32.72, 4.34 for IL-8, respectively compared to the control group (Fig 2).

According to the overall proinflammatory cytokine gene expression results, 100 ng/ml LPS and 1 μg/ml CFS concentrations with 10 ng/ml IFN-ɣ combination were found more effective for the activation of HMC3 microglia cells. Chlamydia and its pathogenic proteins can stimulate the nuclear factor-κB (NF-κB) and MAPK/ERK signaling pathways, as well as an IL-1α-mediated IL-1 receptor type I (IL-1RI)-independent pathway [67, 68]. In addition, the local hypoxic environment formed by the Cpn infection promotes IL-8 release [69]. Interestingly, it was observed that the increase in IL-8 gene expression levels in the presence of CFS was not dose-dependent. This is thought to be due to a mechanism similar to that which causes greater IL-8 secretion in low MOI challenges in the absence of significant TNF-α production [70]. It was discovered that the highest rise in IL-8 was observed at the CFS concentration, which was associated with lower TNF-α expression. As a result, the neuroinflammation model is more effective in mimicking AD brain microenvironment than the LPS model, which has been shown to have elevated levels of IL-8 production by microglia in AD patients [71–73].

#### 3.1.3. Active microglia marker CD11b induction with CFS

**Fig 3:**
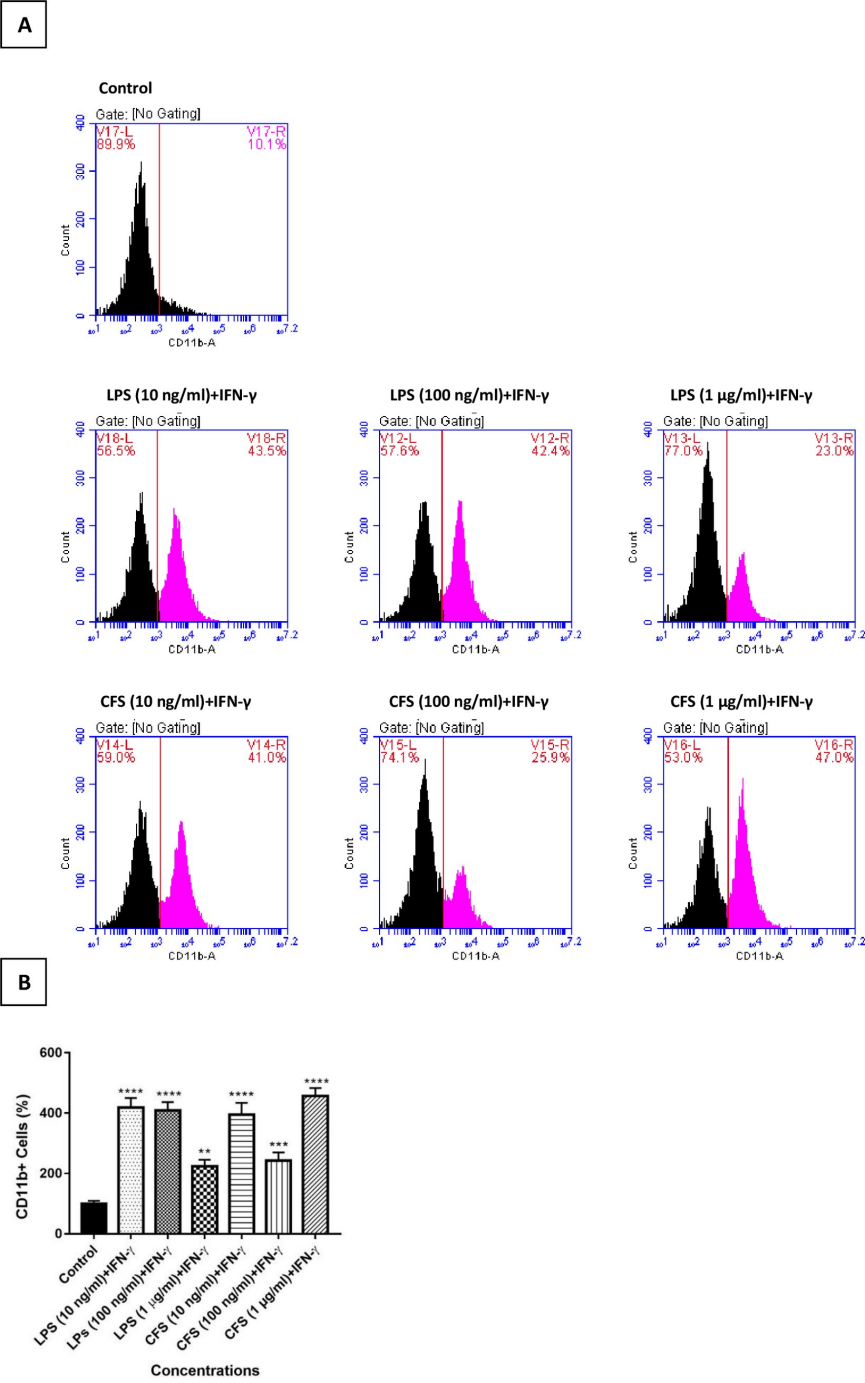
Effects of LPS and CFS (10, 100 ng / ml or 1 μg / ml) + IFN-ɣ (10 ng / ml) concentrations on active microglia marker CD11b antibody levels after 24 hours. (A) CD11b levels in HMC3 cells induced by treatment groups were evaluated using flow cytometry. The control group represents CD11b levels in untreated HMC3 cells. One representative result from three independent repeats is shown. (B) The bar graph shows the % change in CD11b levels in the treatment groups. The CD11b levels of untreated HMC3 cells were used as a control group and the changes in antibody levels of treatment groups were calculated according to this group. The data represents the means ± SDs of three individual experiments (n = 3 for each). One-way ANOVA was used for statistical analysis, followed by a post hoc Tukey’s multiple comparisons test. no difference: p> 0.05; significant difference: *p < 0.05, **p < 0.01, ***p < 0.001, ****p < 0.0001 vs. untreated controls.

During activation, microglia not only secrete various neurotoxic molecules but also express different proteins and surface markers. The increased expression of CD11b is shown to correspond to the intensity of microglial activation in diverse neuroinflammatory disorders [74]. Since microglial activation is represented by increased expression of CD11b, the effects CFS concentrations on microglia activation were compared with LPS by measuring CD11b antibody levels using flow cytometry to determine the CFS concentration to be used in the neuroinflammation model. Since IFN-ɣ stimulates the immune response in cells and enhances the activity of LPS, they are commonly used together in neuroinflammation models. Due to findings of elevated IFN-ɣ levels in numerous neurological disorders, including AD, IFN- ɣ in combination with CFS was used to activate microglia in this study too [75–77]. According to the results, while the control group increased the CD11b antibody levels by 10.1% in 24 hours, the LPS and CFS concentrations of 10, 100 ng/ml or 1 μg/ml in combination with 10 ng/ml IFN-ɣ were increased by 43.5%, 42.4, 23% and 41, 25.9 and 47%, respectively (Fig 3).

Similar to these results, HMC3 microglia cell line has been reported to be CD11b + at the basal level during the resting phase [78–80]. When all the microglia activation related experiment results were examined, activation concentrations to be used in further studies were determined as 100 ng/ml for LPS and 1 μg/ml for CFS in combination with 10 ng/ml IFN-ɣ.

#### 3.1.4. Cell viability of *Chlamydia pneumoniae*-induced neuroinflammation co-culture model

In order to evaluate the cell viability of the cells within the neuroinflammation model, the CI values were recorded using xCELLigence RTCA-DP system. Adhering cells obstruct electrode-culture medium contact and, as a result, electron flow. The degree of this impedance (resistance to alternating current) is called CI and is determined by the number, shape, and size of the cells as well as the strength of cell attachment to the substrate coating the plate [81].

**Fig 4:**
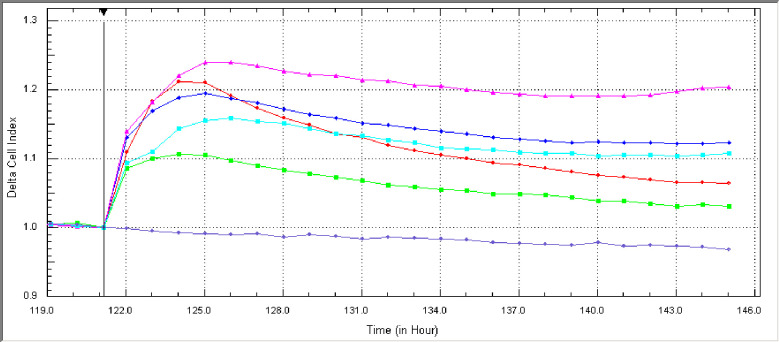
Cell proliferation curves of the differentiated SH-SY5Y cells within the neuroinflammation co-culture model. Effects of control (blue), medium control with no cells (purple), CFS (1 μg/ml) +IFN-ɣ and LPS (100 ng/ml) + IFN-ɣ with (green and red) or without (pink and cyan) inserts on cells within the neuroinflammation co-culture model were drawn based on CI data recorded for 48 hours in the RTCA-DP analysis system.

SH-SY5Y cells were differentiated within the instrument for 5 days and incorporated with E-plate inserts containing CFS+IFN-ɣ or LPS+ IFN-ɣ activated microglia. In parallel, the effects of CFS and LPS on SH-SY5Y cell viability in insert-free wells were also investigated. No difference in cell viability was observed during the application period of the neuroinflammation model, as demonstrated by the plateau-shaped CI data (Fig 4). Prior to treatment, cell index data should be plateaued and close to each other while using this device. The software of the device employs a ratio transformation mechanism, which divides all wells’ CI readouts by the base-time. The transformation reduces the CI values in each well to 1, resulting in a more similar transformed (normalized) CI [53].

### 3.2. Evaluation of the drug concentrations

**Fig 5:**
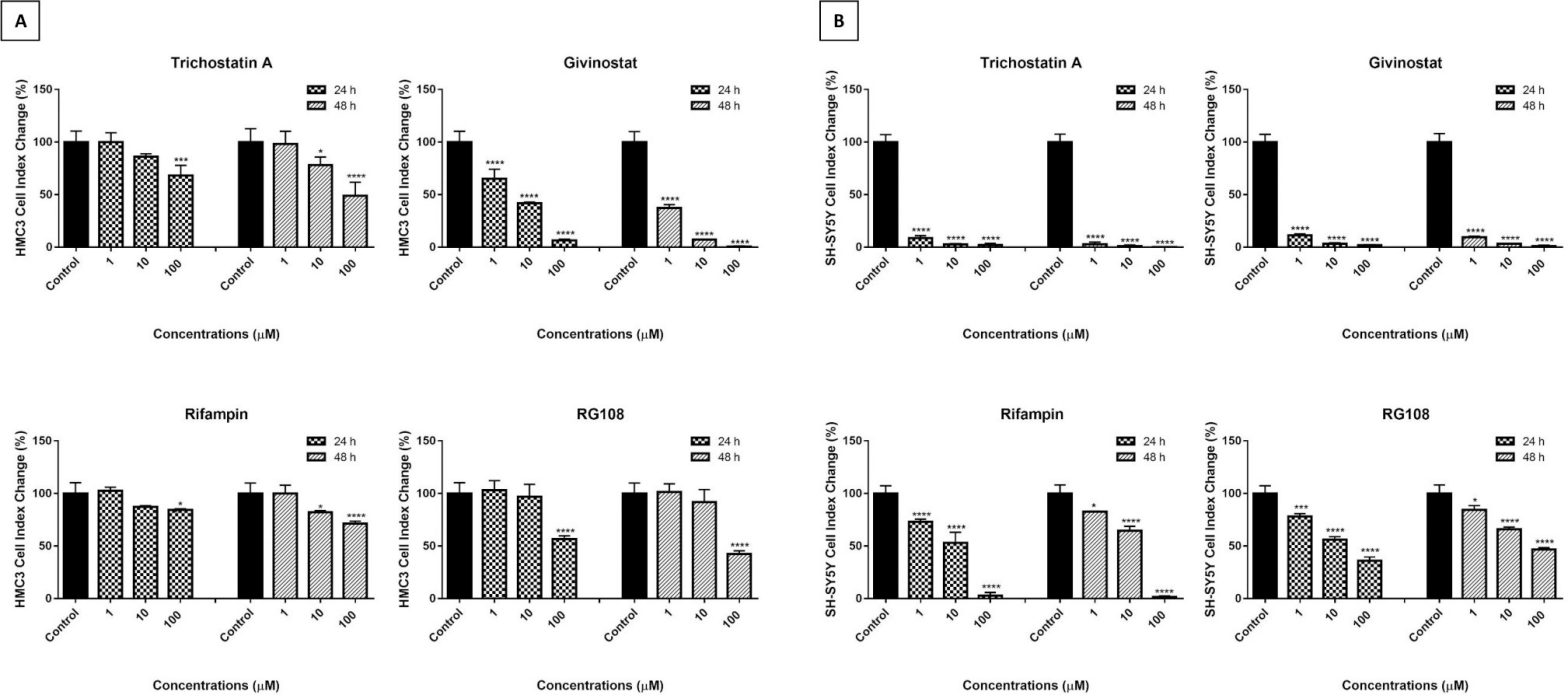
Cell proliferation analysis results of 1, 10 and 100 μM TSA, givinostat, rifampin and RG108 concentrations on HMC3 (A) and SH-SY5Y (B) cells. The effects of treatment groups on HMC3 and differentiated SH-SY5Y cells were monitored real-time for 48 hours and the CI values were recorded. The proliferation slope graph was drawn according to the 24th and 48th hour CI values in the RTCA-DP system. The results are presented as the mean ± SD of three different experiments, n = 8 for each treatment group, significant difference: *p <0.05, **p <0.01, ***p <0.001, ****p <0.0001; no difference: p> 0.05 ns.

In order to determine the non-cytotoxic concentrations of drugs whose neuroprotective effects have been investigated in the neuroinflammation model, the xCELLigence system was used to screen cytotoxicity (Fig 5).

According to the results of the cell proliferation analysis, 1, 10, and 100 M TSA reduced HMC3 cell viability by 0.04, 13.88, and 31.68% at 24 hours, and 1.75%, 21.94, and 51.2 at 48 hours, respectively, compared to the control group. It was increased by 2.43% at 1 μM rifampin and decreased by 12.72% and 15.74% at 10 and 100 μM. At the 48th hour, it decreased by 17.79 and 28.72% with 10 and 100 μM, respectively. 1, 10, and 100 M givinostat reduced HMC3 cell viability by 35.01, 58.19 and 93.58% at 24 hours, and 62.78, 92.67 and 99.29% at 48 hours, respectively, compared to the control group. It was reduced by 3.12 and 43.26% at 24 hours and 8.33 and 57.56% at 48 hours with 10 and 100 μM RG108, respectively.

1, 10, and 100 μM TSA, rifampin, givinostat and RG108 reduced differentiated SH-SY5Y cell viability by 91.34, 97.43 and 97.86%; 30.28, 46.98 and 97.24%; 88.72, 96.85 and 98.06%; 21.98, 43.73 and 64.03% at 24 hours, respectively. At 48 hours, 1, 10, and 100 μM TSA and givinostat concentrations were decreased cell viability by 97.97, 98.9 and 99.79%; 90.56, 96.9 and 98.77%, respectively.1, 10 and 100 μM rifampin and RG108 were reduced SH-SY5Y cell viability by 17.16, 25 and 98.61%; and 15.47, 33.89 and 53.43% at 48 hours, respectively.

The IC_50_ concentrations of treatment groups on HMC3 (S2 Fig) and SH-SY5Y (S3 Fig) cells were calculated using the CI values at 24 and 48 hours (Table 1). Through comparing the average IC_50_ concentrations, the non-cytotoxic concentration to be used in future studies was calculated to be 100 nM, which is below the lowest value seen in all cells.

**Table 1:** IC_50_ values of SH-SY5Y and HMC3 cells.

Treatment Groups	IC_50_ Values			
	24h		48h	
	SH-SY5Y	HMC3	SH-SY5Y	HMC3
**TSA**	596 nM	172 nM	312 nM	156 nM
**Givinostat**	2.78 μM	11.8 μM	1.42 μM	539 nM
**RG108**	1.49 μM	>400 μM	9.6 μM	>400 μM
**Rifampin**	>400 μM	33.2 μM	64.4 μM	31 μM

Cells were treated with 1, 10 and 100 μM TSA, givinostat, rifampin, and RG108 concentrations and the IC_50_ values were calculated according to the CI values at 24th and 48th hours in the analysis software of the instrument.

**Fig 6:**
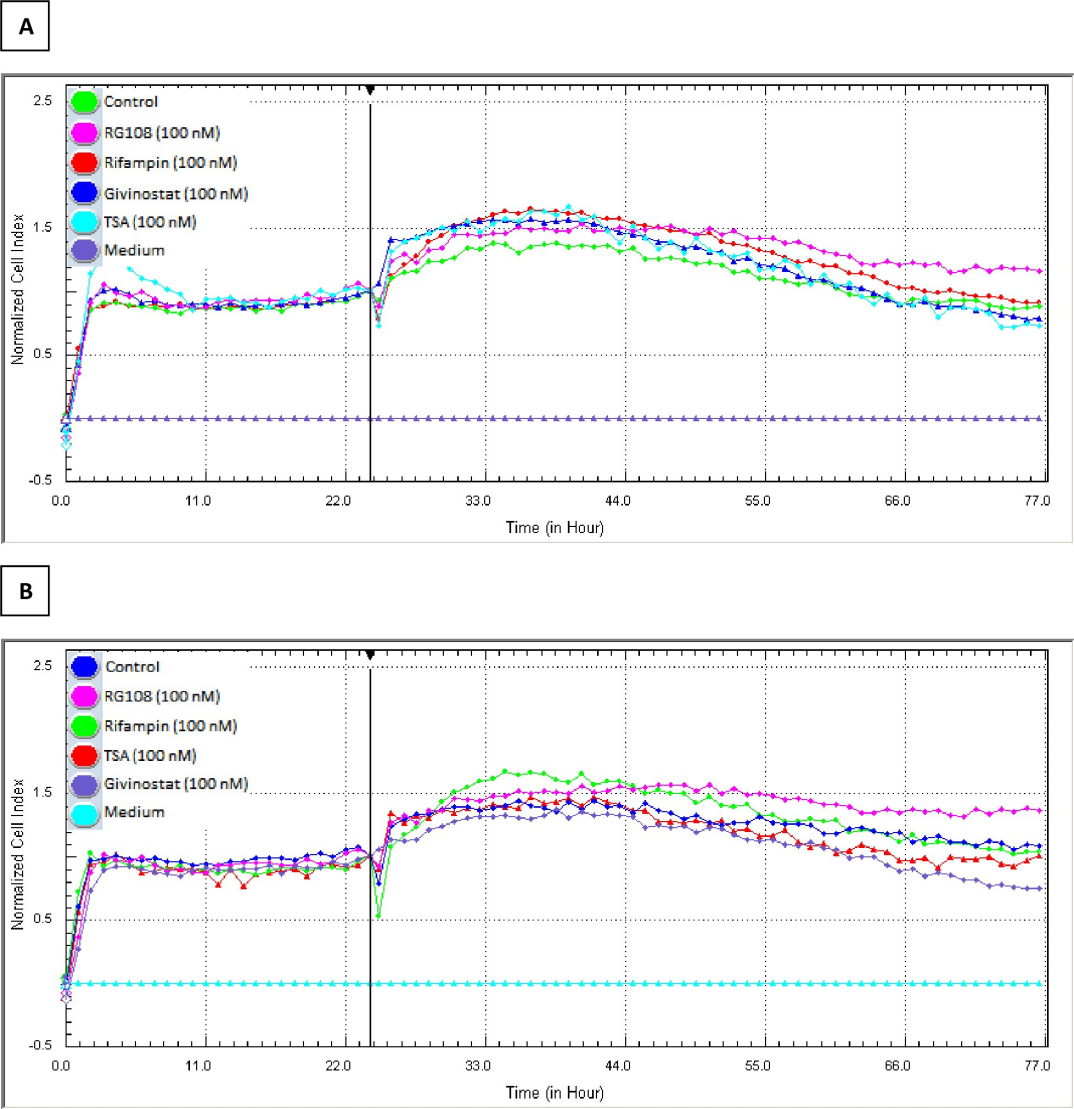
Effects of the treatment groups on HMC3 (A) and differentiated SH-SY5Y (B) cells at 100 nM concentrations. The treatment groups whose neuroprotective effects were tested have no effect on HMC3 and SH-SY5Y cell viability at the chosen concentration (100 nM), as shown by the plateau image of the CI data. 24 hours after seeding the previously differentiated SH-SY5Y and HMC3 cells onto the e-plate, the device was stopped and 100 nM drug concentrations were applied, and the effects were monitored in real time for 48 hours. Data was confirmed in three independent experiments, n = 3 for each treatment group.

Through comparing the average IC_50_ concentrations, the non-cytotoxic concentration to be used in future studies was calculated to be 100 nM, which is below the lowest value seen in all cells. In addition, using the same instrument, it was shown that the 100 nM concentration chosen as the non-cytotoxic concentration did not have a cytotoxic effect on the viability of HMC3 and differentiated SH-SY5Y cells (Fig 6).

### 3.3. Investigation of the effects of CFS, LPS and epigenetic modifying drugs in the presence of Aβ

#### 3.3.1. Evaluation of neuroprotective effects against Aβ-induced neurotoxicity

**Fig 7:**
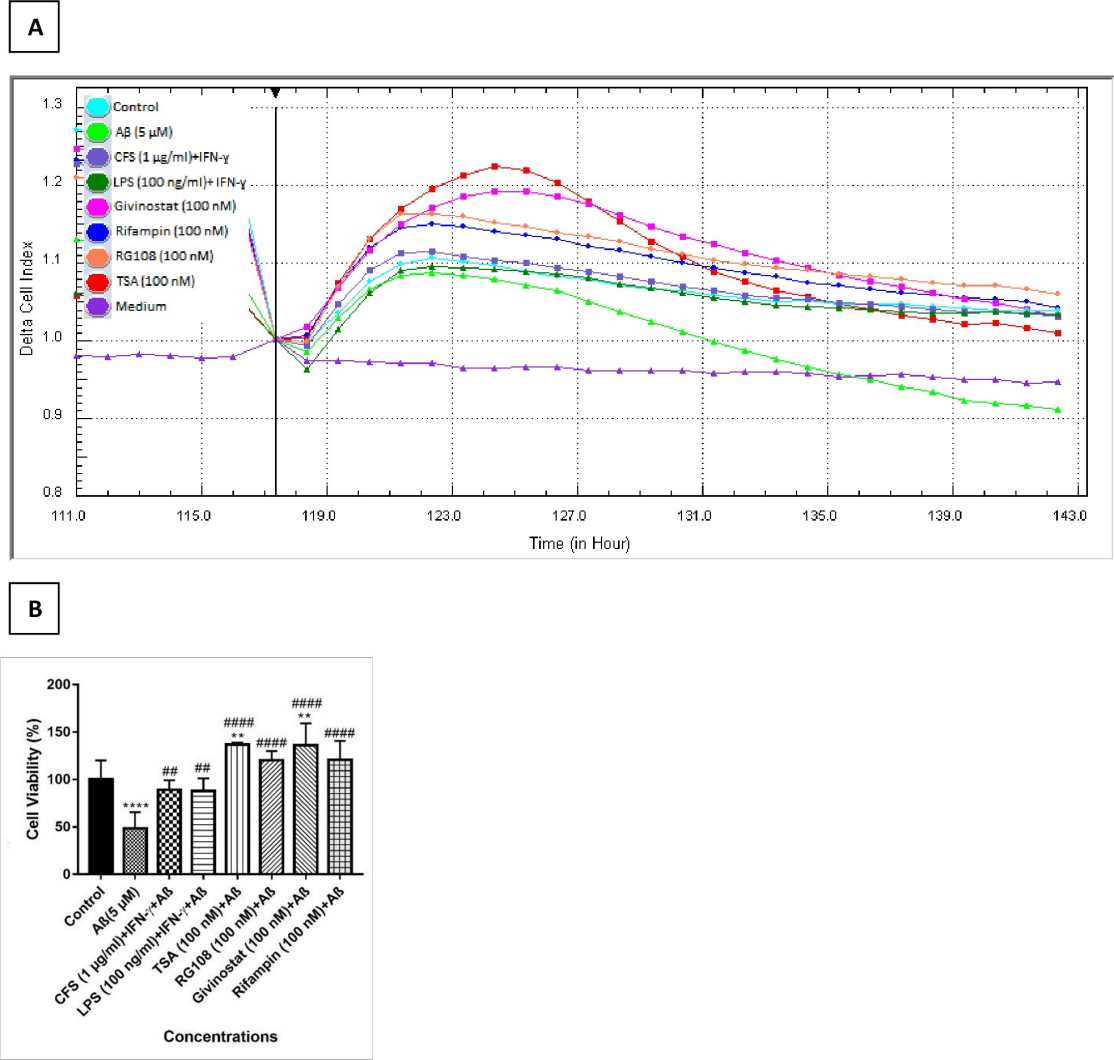
Effects of the treatment groups on differentiated SH-SY5Y cell viability in the presence of 5 μm Aβ. (A) After 24 hours of incubation with treatment groups, real-time CI values were monitored to investigate the neuroprotective effects of the treatment groups against 5 μm Aβ toxicity (n = 4). (B) The slope graph is drawn according to the average CI data at 0^th^, 12^th^ and 24^th^ hours after Aβ application to SH-SY5Y cells for the neurotoxicity analysis. The results are presented as the mean ± SD of three different experiments, n = 4 for each treatment group, no difference: p> 0.05 ns; significant difference: **p <0.01 and ****p <0.0001 according to the control group; significant difference: ^##^p <0.01 and ^####^ p <0.0001 according to the Aβ group).

The neuroprotective effects of epigenetic modifying drugs as well as the effects LPS and CFS in the presence of Aβ were investigated in this study. After 5 days of differentiation in the real-time cell analyzer, Cells in wells without E-plate inserts were incubated with treatment groups for 24 hours. The protective effects of the applied drug concentrations against Aβ neurotoxicity were determined by comparing the CI data obtained from the device after the existing medium in the wells and the medium containing aggregates of 5 μM Aβ (1–42) peptides were changed. According to the results, CI values decreased to 48.5%, 89.1 and 87.9%, respectively, in Aβ (5 μM), CFS + IFN-γ (1 μg/ml) and LPS + IFN-γ (100 ng/ml) groups; TSA, Givinostat, Rifampin and RG108 (100 nM) increased CI values to 137, 136.2, 120.7 and 120.7%, respectively, compared to the control group (Fig 7). S4 Fig shows the baseline delta CI and WST-1 data that validate the real-time cell analysis results.

#### 3.3.2. Evaluation of the effects of treatment groups on Aβ-induced cell death

**Fig 8:**
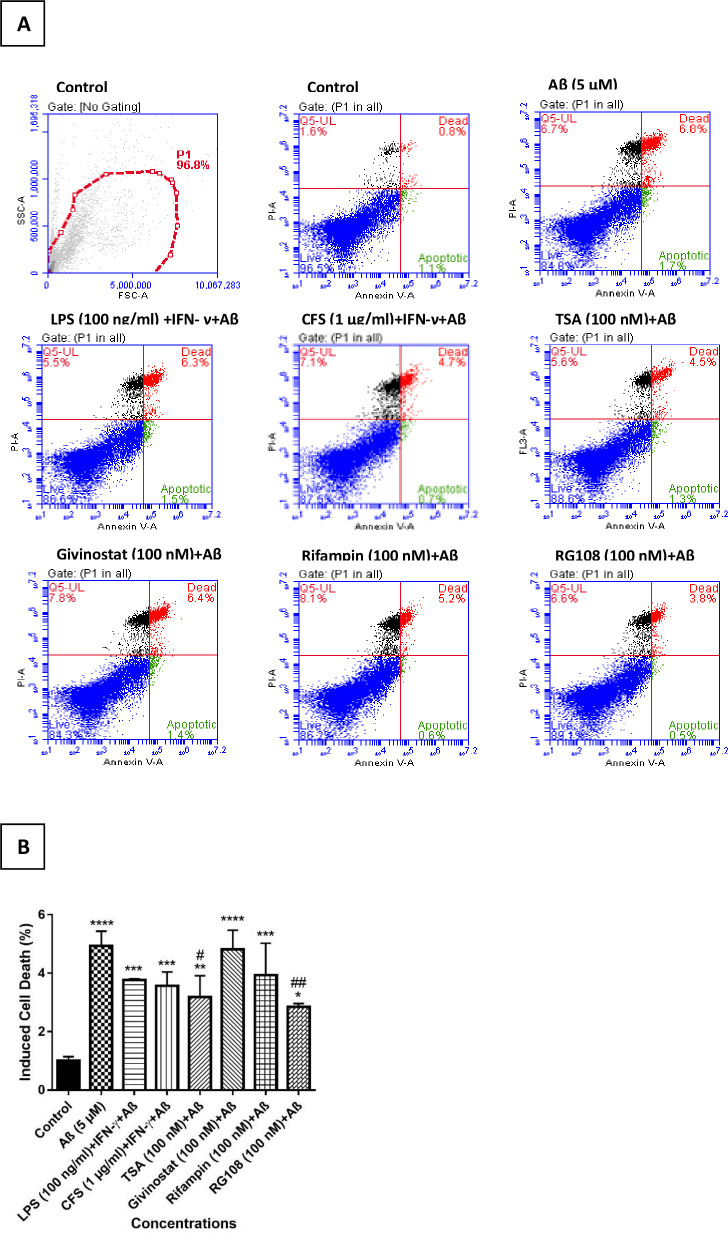
Effects of treatment groups on Aβ-induced cell death (apoptosis+necrosis) in the presence of 5 μM Aβ. (A) The rate of apoptosis/necrosis was measured by flow cytometry using Annexin V/PI staining. Blue dots represent living cells, black dots represent necrotic cells, red and green dots represent cells undergoing late and early apoptosis, respectively. The percentages of cells in each quadrant are given. The analysis was performed with the BD FACSDiva software. Untreated cells were used as control. (One representative result from three independent repeats is shown), (B) The bar graph represents the percentages of cell death induced by Aβ. The graph was plotted by averaging the apoptotic+necrotic cells. The data represents the means ± SDs of three individual experiments (n = 3 for each). One-way ANOVA was used for statistical analysis, followed by a post hoc Tukey’s multiple comparisons test. no difference: p> 0.05; significant difference: *p < 0.05, **p < 0.01, ***p < 0.001, ****p < 0.0001 according to the control group; significant difference: #p <0.05, ##p<0.01 according to the Aβ group.

Apoptotic effect (early apoptosis + late apoptosis) of control, Aβ, LPS + IFN-γ, CFS + IFN-γ, TSA, givinostat, rifampin and RG108 groups were 1.9, 8.5, 7.8, 5.4, 5.8, 7.8, 5.8 and 4.3%, respectively. Necrotic effects of the treatment groups were found as 1.6, 6.7, 5.5, 7.1, 5.6, 7.8, 8.1 and 6.6%, respectively. According to these data, only LPS + IFN-γ and TSA group reduced the necrotic effect compared to the Aβ group. The effect of control, Aβ, LPS + IFN-ɣ, CFS + IFN-ɣ, TSA, givinostat, rifampin, and RG108 treatment groups on the percentage of living cells was observed to be 96.5, 84.8, 86.8, 87.5, 88.6, 84.3, 86.2, and 89.1%, respectively. LPS + IFN-γ, CFS + IFN-γ, TSA, Rifampin and RG108 treatment groups increased cell viability by 2, 2.7, 3.8, 1.4 and 4.3%, respectively, compared to the Aβ positive control group. Givinostat reduced it by 0.5%. Cell viability increase against apoptotic + necrotic effect of Aβ was found significant only in 100 nM TSA (p <0.05^#^) and RG108 (p <0.01^##^) treatment groups (Fig 8).

#### 3.3.3. Evaluation of the effects of treatment groups on ROS

**Fig 9:**
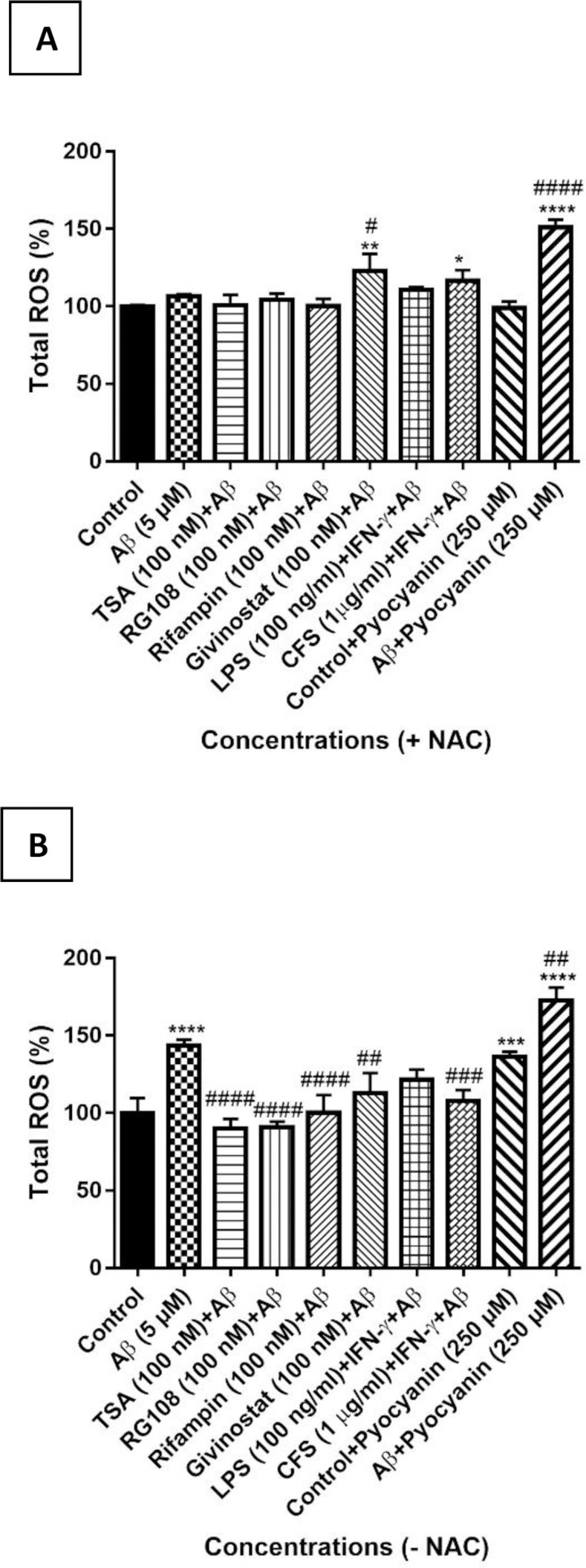
Percentage graph of the total amount of ROS. ROS levels were determined by ELISA method according to the treatment groups (with (A) or without (B) the presence of ROS inhibitor 5 mM N-acetyl cysteine (NAC)). The data represents the means ± SDs of three individual experiments (n = 4 for each). One-way ANOVA was used for statistical analysis, followed by a post hoc Tukey’s multiple comparisons test. no difference: p> 0.05 ns; significant difference: *p <0.05, ** p <0.01, *** p <0.001 and ****p <0.0001 according to the control group; ^#^p <0.05, ^##^p <0.01, ^###^p <0.001 and ^####^p <0.0001 according to the Aβ positive control group.

ROS quantities were measured to evaluate whether the treatment groups provided protection against Aβ-induced ROS production of SH-SY5Y cells used in the neurotoxicity model. According to the results, ROS levels increased by 43.58% only in the presence of Aβ. In the presence of the ROS inducer pyocyanin, ROS levels increased by 36.49% in the control group. ROS production increased by 72.71% in the presence of pyocyanin in the Aβ group as well. This increase in ROS amount compared to the control group was significant only in pyocyanin (p <0.001 ***), Aβ (p <0.0001 ****) and Aβ + pyocyanin (p <0.0001 ****) groups. In cells treated with compounds before exposure to Aβ, the total amount of ROS with TSA and RG108 decreased by 9.85 and 9.03%, respectively, compared to the control group; In Rifampin, Givinostat, LPS / IFN-γ and CFS / IFN-γ groups, the total amount of ROS increased by 0.43, 13.09, 21.67 and 7.89%, respectively, compared to the control group, but these changes were not significant. When the total ROS amount was examined according to the Aβ group, it decreased by 53.43, 43.14, 30.49, 52.61, 21.91 and 35.69% in the TSA, rifampin, givinostat, RG108, LPS + IFN-ɣ and CFS + IFN-γ groups, respectively. This decrease in the ROS levels compared to the Aβ group was found to be significant in TSA, RG108 and Rifampin (p <0.0001 ^####^), Givinostat and CFS + IFN-γ (p <0.01 ^##^) groups (Fig 9).

When the cells were incubated for 30 minutes with the ROS inhibitor NAC before the measurement, the total amount of ROS in the Aβ group increased by 6.62% compared to the control group. SH-SY5Y cells treated with TSA, rifampin, givinostat, RG108, LPS + IFN-γ and CFS + IFN-γ groups prior to Aβ exposure increased by 0.7, 0.25, 22.87, 4.29, 10.54 and 16.52%, respectively, but this increase was found to be significant in only Givinostat (p <0.01 **) and CFS + IFN-γ (p <0.5 *) groups. This data can be interpreted as ROS activation by Givinostat and CFS + IFN-γ works through a different pathway with the ROS inhibitor NAC. In the presence of the ROS inducer pyocyanin, the amount of ROS decreased by 0.85% and increased by 51.07% in the presence of Aβ (p <0.0001 ****) (Fig 9). In the presence of ROS inhibitor NAC, there was no significant increase in these groups. The increase in ROS in the presence of Aβ + pyocyanin could not be inhibited by NAC can be interpreted as that Aβ and pyocyanin might have potentiated the effects of each other.

## 4. Discussion

*Chlamydiae* have a biphasic life cycle with two functional and morphologically distinct forms: an infectious and metabolically inert elementary body (EB) that promotes binding and transmission to the target host cell; and reticulate body (RB), a non-infectious and metabolically active intracellular form that proliferates before being reorganized into an elementary body and released via cell lysis [82]. Unlike other members of the *Chlamydia* family, Cpn is difficult to propagate, isolate and detect [83]. Therefore, lyophilized CFS obtained from the infected cell culture was used to develop an *in vitro* Cpn-induced neuroinflammation model. CFS can be employed in a similar way to that of commercially available lyophilized inductive agents. Similar studies exist in the literature [84–86], but this is the first time Cpn has been used in a model. The ability to change the concentration with lyophilized CFS without having to return to the inoculation step each time when working with Cpn is the model’s major benefit. Another advantage of the CFS-induced neuroinflammation model over the LPS-induced model is that it better mimics elevated IL-8 levels in the AD neuroinflammation microenvironment. It was reported that IL-8 may play a role in boosting pro-inflammatory reactivity in AD, according to studies on cultured human microglia. In comparison to other inflammatory mediators, chemokine family members showed the most increased gene expression. The expression of IL-8 was the one that was upregulated the most [73, 87].

The use of IFN-ɣ combination with Cpn for microglia activation in this study is primarily due to reports of elevated levels of IFN-ɣ in various neurological disorders and animal models, such as stroke, multiple sclerosis, and AD [66, 88–92]. Similarly, since IFN-ɣ stimulates the immune response in cells and enhances the inducing activity of LPS, they are also used together in inflammation models [75–77]. A study of peripheral blood mononuclear cells yielded similar findings to our cytokine gene expression results within the model. TNF-α and IL-1β cytokine production increased five and two-fold, respectively, after 24 hours of incubation with sonicated 10^4^ IFU/ml Cpn, relative to the control sample, while IL-6 and IL-8 production increased nearly 40-fold [93]. In another study, a significant increase in the expression of 17 genes, mainly TNF-α and genes associated with the innate or adaptive immune system, was observed in a study in which THP-1 monocyte cells were infected with 1 MOI Cpn [94]. In addition to the cytokine analysis, CD11b antibody levels were determined in the model to assess microglial activation. Flow cytometry is used in many experiments to assess CD11b+ cells as an activation marker [95–98]. The HMC3 cell line was previously stated to be CD11b + at the basal level in the resting phase, similar to our result of 10.1% positive antibody levels in the 24 hours measured in the control group [79, 80].

Amyloidogenesis and tau protein hyperphosphorylation contribute to neurotoxicity and neuronal cell death in Alzheimer’s disease caused by the aggregation of Aß. However, clinical trial failures targeting Aß in the brain point to the need for new ideas to explain AD development and novel intervention targets to prevent and cure the disease. Many experimental data suggest that a microbial community plays a role AD pathogenesis. As a result, hypotheses such as "infection" and "antimicrobial protection" have been presented to explain AD pathogenesis. Because Aß is back in the forefront, but this time as a tool to neuroinflammation rather as a main character, the old amyloid hypothesis is naturally included into the new thoughts [99]. This perspective was taken into consideration when investigating the effects of CFS and LPS in the presence of Aß, as well as when evaluating the neuroprotective effects of epigenetic modifying drugs on Aβ-induced neurotoxicity. According to the results, although cell viability decreased in the Aβ, CFS and LPS groups relative to the control group, cell viability increased significantly with TSA, givinostat, rifampin, and RG108, and these groups provided neuroprotection against Aβ neurotoxicity *in vitro*. In the absence of activated microglia, there was no significant neurotoxic effect in the LPS and CFS groups when Aβ was present. It has been reported that LPS alone had no effect on SH-SY5Y cell viability [100, 101] and a low dose of LPS pretreatment causes a hyporesponsive state to a subsequent secondary challenge [102, 103].

The role of epigenetic modifications in the pathogenesis of neurodegenerative disorders is becoming increasingly evident as research advances. Through cellular-level processes like neurogenesis and DNA repair, these changes impact broad and complicated processes like brain development, memory formation, motor control, and cognition. As a result, it has been shown that an inconsistency of epigenetic processes influences the evolution of certain neurological diseases, regardless of genetic code anomalies [104]. There are similar findings in the literature that corroborate the neuroprotective action seen in treatment groups. Rifampin has been shown to induce neuroprotection in AD by increasing Aβ clearance by upregulating low density lipoprotein receptor-related protein 1 (LRP-1) and P-glycoprotein (P-gp) [44]. In mutated COS-7 cells, which express amyloid precursor protein and increase Aβ oligomerization, rifampin has been shown to minimize Aβ, tau, and α-synuclein aggregation [45]. Recently, TSA treatment was found to reduce Aβ plaques and soluble Aβ oligomers in the brain, as well as improve learning and memory behaviors in APP/PS1 mice [105]. Inhibiting DNMT catalytic activity with small molecules such as RG108 has been shown to protect motor neurons from excessive DNA methylation and apoptosis [106]. There is no published research on givinostat’s neuroprotective effects. Givinostat is an acridine derivative and due to their simple structure and strong ligand ability, acridine derivatives can be considered an important ligand for the design of multitarget-directed agents against Aβ associated with AD pathology [107]. Additionally, givinostat could be an ideal candidate for AD therapy due to its anti-inflammatory effects [108–110].

Chronic bacterial or viral toxic products, which result in the presence of excess reactive oxygen species and culminate in pathologic alterations, are an appealing concept for the cause or development of neurological disease. Infection with Cpn has been demonstrated to cause ROS generation in all cells, resulting in oxidative stress [111–115]. According to our results, it was found that TSA, RG108, givinostat and rifampin had the capacity to protect neurons by lowering intracellular production of Aβ-induced ROS. It is hypothesized that the two exopolysaccharides present in bacterial cells, LPS and Cpn, generate intracellular ROS by distinct pathways, based on results obtained using the ROS inhibitor NAC and the ROS inducer pyocyanin. Although there are studies on the effects of rifampin [116–118], TSA [119–121] and RG108 [122] on oxidative stress in the literature, this study is the first study on the inhibitory effect of givinostat on Aβ-induced ROS induction.

Neuroinflammation has become a popular topic in neurological research in recent years, with a focus on the activation and inflammatory response of astrocytes and microglia, making neuron and glial cell models important study tools for neurodegenerative diseases [123]. Although the Cpn-induced neuroinflammation model is one of these tools, its utilization has limitations like other *in vitro* co-culture methods. The model’s design with immortalized cells and in two-dimensions (2D) may lead to differences in the one-to-one mimicking of cellular responses when compared to experiments performed *in vivo* or with primary cells. This model lacks the extracellular matrix structure of 3D models, and the cells grow on a flat surface, allowing all cells to contact nutrients and drugs. Despite these limitations, it is a very useful model for evaluating preliminary drug screening because of its rapidity, low cost, simple procedure, reproducibility, and compatibility with existing analyzers.

## 5. Conclusion

In conclusion, a modified LPS-like model has been established with CFS that can be used in Cpn-induced *in vitro* neuroinflammation models. TSA, RG108, givinostat, and rifampin all demonstrated neuroprotective effects in this novel model as well as in Aβ-induced neurotoxicity, according to this study. It is believed that the findings from this research will be preliminary candidates for new drug combinations or therapies to address existing medications that have the potential to be effective in managing epigenetic and inflammatory mechanisms that may be found in AD and similar neurodegenerative diseases. The data from this study could serve as an alternative approach to treating AD, a degenerative brain disease for which there are limited therapeutic options. Further research at the molecular and clinical levels is needed to fully develop this treatment option.

## Supporting information

S1 Fig Cpn inclusion bodies (left: 2.5x, right: 10x). Cpn was propagated using the HEp-2 cell line.
(DOCX)

S2 Fig Cell proliferation analysis results of HMC3 cells. A: Real-time monitoring of the effects of treatment groups on HMC3 cell viability for 48 hours using the xCELLigence instrument. The CI values were recorded every 1 h with the instrument, which appeared as each dot on the graph based on the electrical impedance. B: IC_50_ values of HMC3 cells. Cells were treated with 1, 10 and 100 μM TSA, givinostat, rifampin, and RG108 concentrations and the IC_50_ values were calculated according to the CI values at 24th (red) and 48th (green) hours in the analysis software of the instrument. Data was confirmed in three independent experiments, n = 8 for each treatment group.
(DOCX)

S3 Fig Cell proliferation analysis results of SH-SY5Y cells. A: Real-time monitoring of the effects of treatment groups on SH-SY5Y cell viability for 48 hours using the xCELLigence instrument. The CI values were recorded every 1 h with the instrument, which appeared as each dot on the graph based on the electrical impedance. B: IC_50_ values of SH-SY5Y cells. Cells were treated with 1, 10 and 100 μM TSA, givinostat, rifampin, and RG108 concentrations and the IC_50_ values were calculated according to the CI values at 24th (red) and 48th (green) hours in the analysis software of the instrument. Data was confirmed in three independent experiments, n = 8 for each treatment group.
(DOCX)

S4 Fig Data supporting neuroprotective activity results with the xCELLigence device. (A) Baseline delta CI correction results to eliminate the effects of amyloid beta oligomers on CI data. In this analysis, it was investigated whether the increase in cell viability in treatment groups was due to electrical impedance from Aβ oligomers. It is determined by subtracting the CI values of the amyloid beta group defined by the device from all CI values. (B) WST-1 cell viability assay results. Parallel to experiments using a real-time cell analyzer, WST-1 analysis was carried out in 96-well plates. The slope graph was drawn according to the colorimetric analysis results 24 hours after Aβ application to SH-SY5Y cells for neurotoxicity. One-way ANOVA was used for statistical analysis, followed by a post hoc Tukey’s multiple comparisons test. The results are presented as the mean ± SD of three different experiments, n = 8 for each treatment group, no difference: p> 0.05 ns; significant difference: ***p <0.001 and ****p <0.0001 according to the control group; significant difference: ^####^ p <0.0001 according to the Aβ group).
(DOCX)

S1 Table IL-1β, TNF-α, IL-8 and ACTB primer sequences. (DOCX)

S2 Table RT-PCR cycling conditions. (DOCX)
