# Supplementary figures and images for: Neuroprotective effects of some epigenetic modifying drugs’ on Chlamydia pneumoniae-induced neuroinflammation: A novel model
Source: PLoS One. 2021 Nov 30;16(11):e0260633. doi: 10.1371/journal.pone.0260633 (PMC8631675; doi:10.1371/journal.pone.0260633)

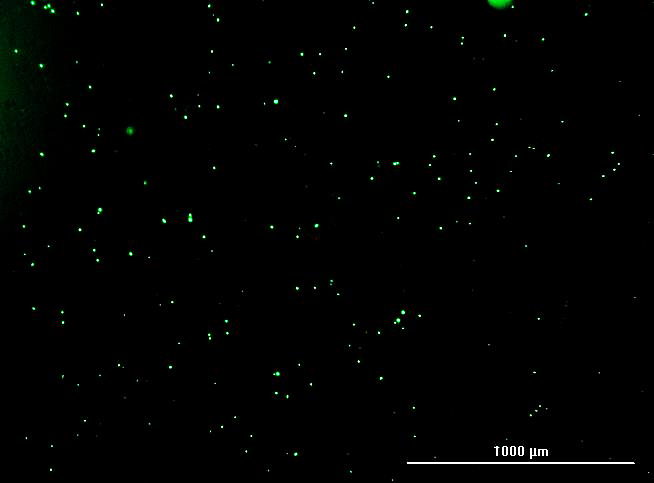

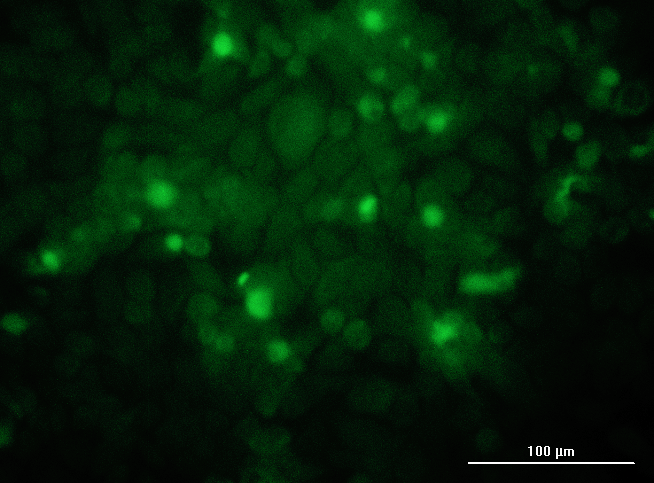


**S1 Fig. Cpn inclusion bodies (left: 2.5x, right: 10x).** Cpn was propagated using the HEp-2 cell line.

Supplement: S1 Fig — Cpn was propagated using the HEp-2 cell line. (DOCX) [file pone.0260633.s001.docx]
