# Supplementary material for: Neuroprotective effects of some epigenetic modifying drugs’ on Chlamydia pneumoniae-induced neuroinflammation: A novel model
Source: PLoS One. 2021 Nov 30;16(11):e0260633. doi: 10.1371/journal.pone.0260633 (PMC8631675; doi:10.1371/journal.pone.0260633)
Supplement: S2 Fig — A: Real-time monitoring of the effects of treatment groups on HMC3 cell viability for 48 hours using the xCELLigence instrument. The CI values were recorded every 1 h with the instrument, which appeared as each dot on the graph based on the electrical impedance. B: IC50 values of HMC3 cells. Cells were treated with 1, 10 and 100 μM TSA, givinostat, rifampin, and RG108 concentrations and the IC50 values were calculated according to the CI values at 24th (red) and 48th (green) hours in the analysis software of the instrument. Data was confirmed in three independent experiments, n = 8 for each treatment group. (DOCX) [file pone.0260633.s002.docx]

**A**


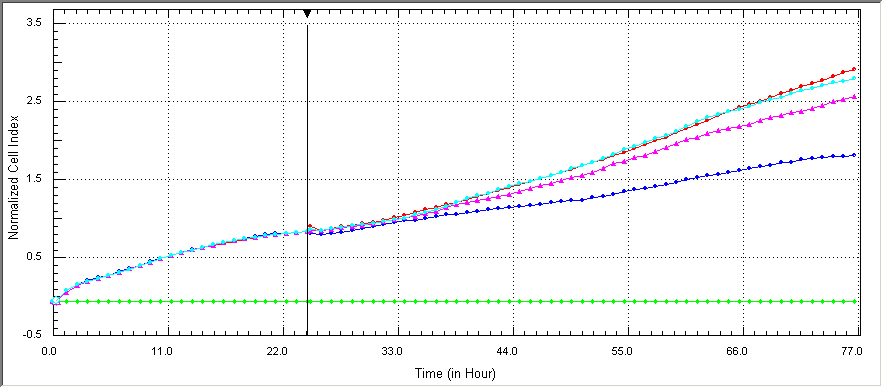

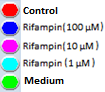

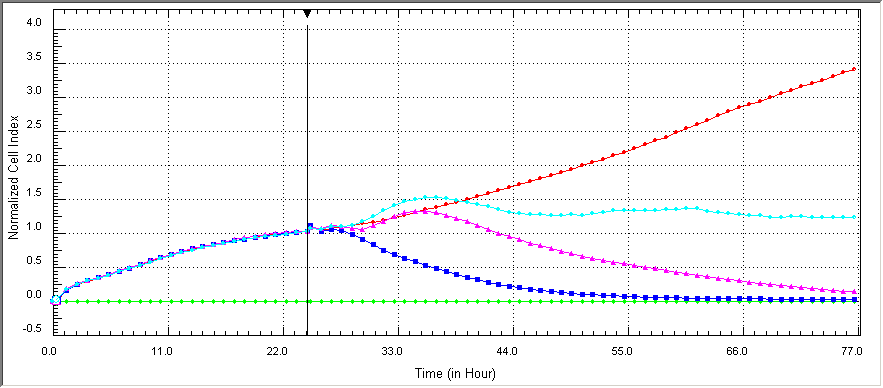

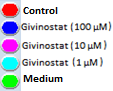

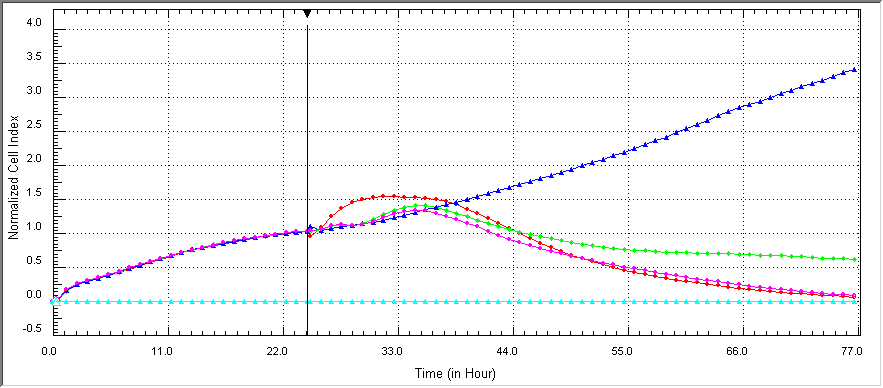

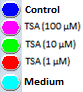

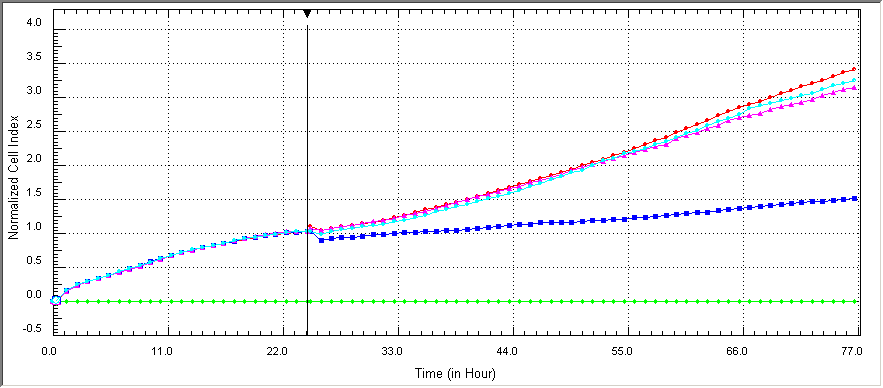

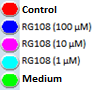


**B**


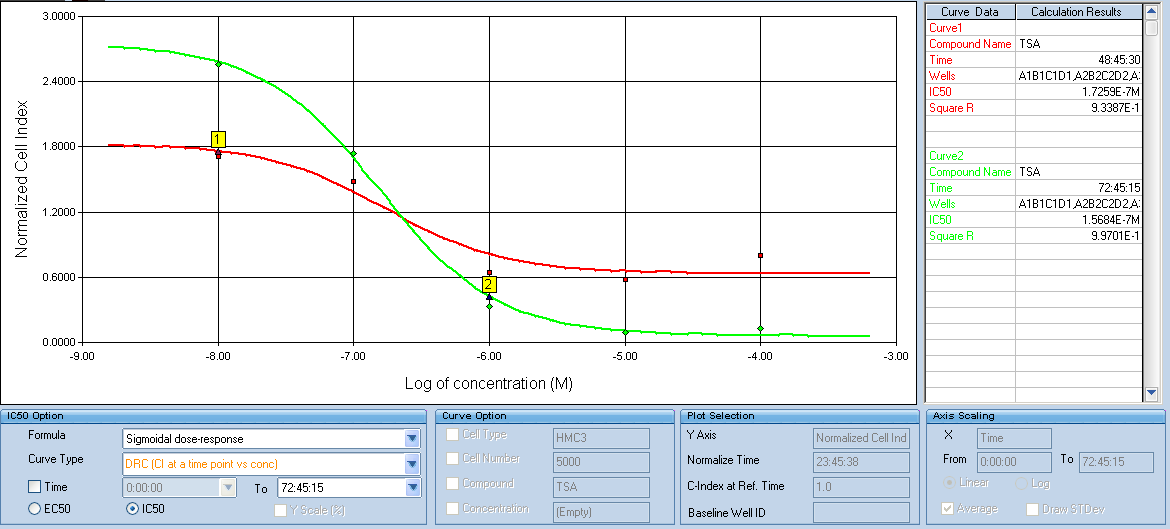


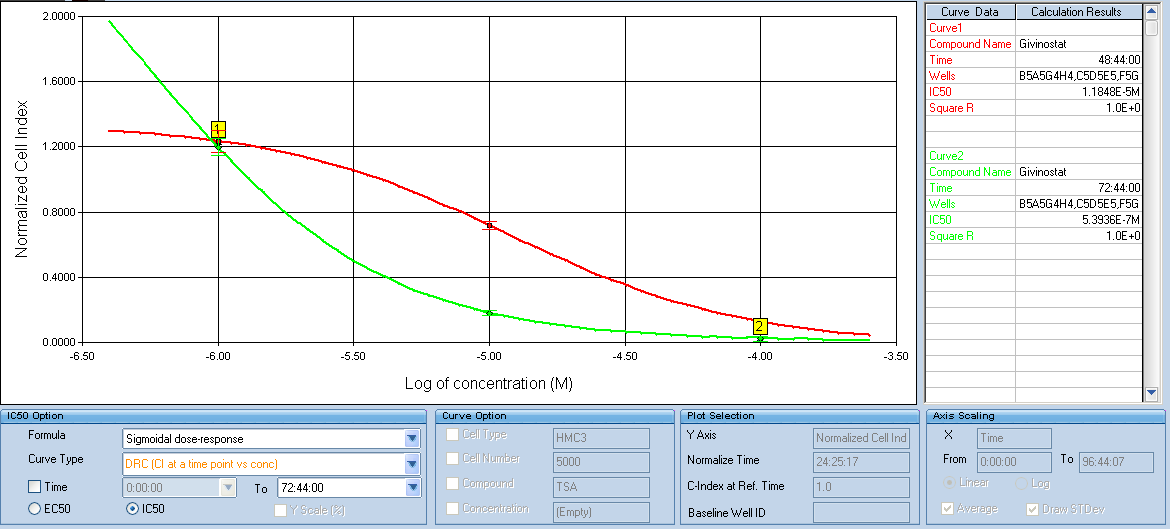


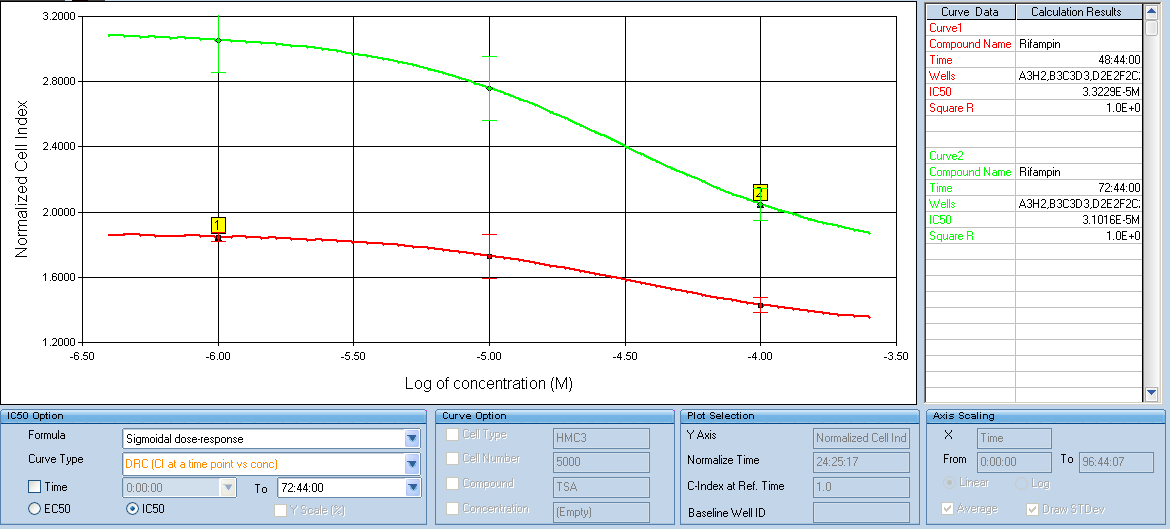


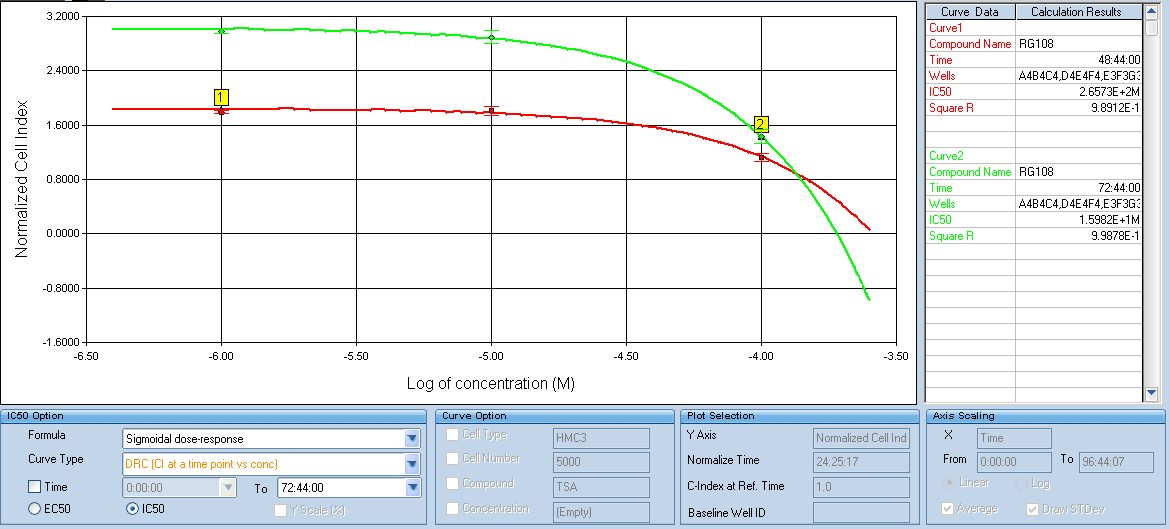


**S2 Fig. Cell proliferation analysis results of HMC3 cells.** A: Real-time monitoring of the effects of treatment groups on HMC3 cell viability for 48 hours using the xCELLigence instrument. The CI values were recorded every 1 h with the instrument, which appeared as each dot on the graph based on the electrical impedance. B: IC_50_ values of HMC3 cells. Cells were treated with 1, 10 and 100 µM TSA, givinostat, rifampin, and RG108 concentrations and the IC_50_ values were calculated according to the CI values at 24th (red) and 48th (green) hours in the analysis software of the instrument. Data was confirmed in three independent experiments, n=8 for each treatment group.
