# Supplementary material for: Neuroprotective effects of some epigenetic modifying drugs’ on Chlamydia pneumoniae-induced neuroinflammation: A novel model
Source: PLoS One. 2021 Nov 30;16(11):e0260633. doi: 10.1371/journal.pone.0260633 (PMC8631675; doi:10.1371/journal.pone.0260633)
Supplement: S4 Fig — (A) Baseline delta CI correction results to eliminate the effects of amyloid beta oligomers on CI data. In this analysis, it was investigated whether the increase in cell viability in treatment groups was due to electrical impedance from Aβ oligomers. It is determined by subtracting the CI values of the amyloid beta group defined by the device from all CI values. (B) WST-1 cell viability assay results. Parallel to experiments using a real-time cell analyzer, WST-1 analysis was carried out in 96-well plates. The slope graph was drawn according to the colorimetric analysis results 24 hours after Aβ application to SH-SY5Y cells for neurotoxicity. One-way ANOVA was used for statistical analysis, followed by a post hoc Tukey’s multiple comparisons test. The results are presented as the mean ± SD of three different experiments, n = 8 for each treatment group, no difference: p> 0.05 ns; significant difference: ***p <0.001 and ****p <0.0001 according to the control group; significant difference: #### p <0.0001 according to the Aβ group). (DOCX) [file pone.0260633.s004.docx]

**A**


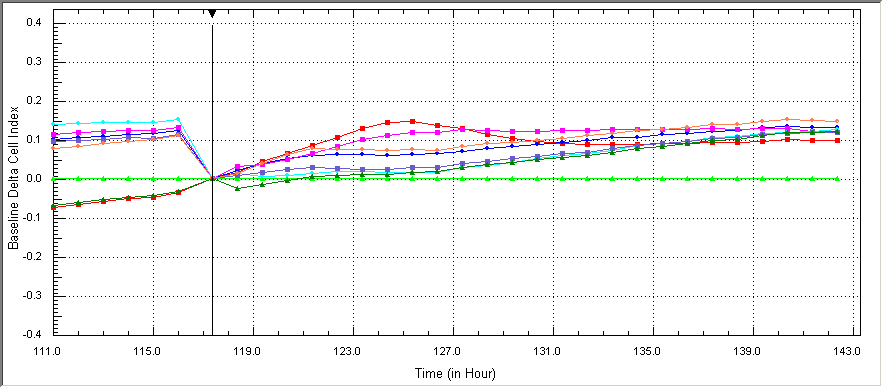

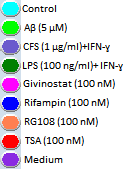


**B**


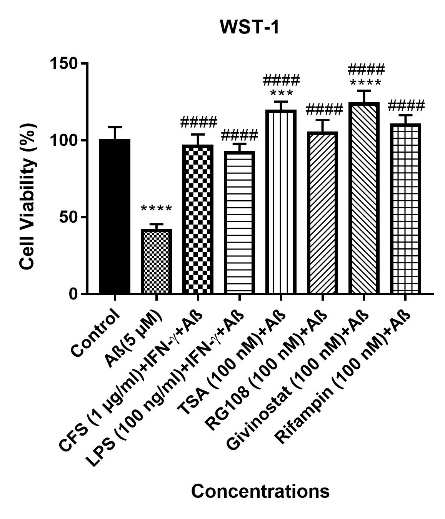


**S4 Fig. Data supporting neuroprotective activity results with the xCELLigence device.** (A) Baseline delta CI correction results to eliminate the effects of amyloid beta oligomers on CI data. In this analysis, it was investigated whether the increase in cell viability in treatment groups was due to electrical impedance from Aβ oligomers. It is determined by subtracting the CI values of the amyloid beta group defined by the device from all CI values. (B) WST-1 cell viability assay results. Parallel to experiments using a real-time cell analyzer, WST-1 analysis was carried out in 96-well plates. The slope graph was drawn according to the colorimetric analysis results 24 hours after Aβ application to SH-SY5Y cells for neurotoxicity. One-way ANOVA was used for statistical analysis, followed by a post hoc Tukey's multiple comparisons test. The results are presented as the mean ± SD of three different experiments, n=8 for each treatment group, no difference: p> 0.05 ns; significant difference: ***p <0.001 and ****p <0.0001 according to the control group; significant difference: ^####^ p <0.0001 according to the Aβ group).
