# Supplementary material for: Neuroprotective effects of some epigenetic modifying drugs’ on Chlamydia pneumoniae-induced neuroinflammation: A novel model
Source: PLoS One. 2021 Nov 30;16(11):e0260633. doi: 10.1371/journal.pone.0260633 (PMC8631675; doi:10.1371/journal.pone.0260633)
Supplement: S1 Table — (DOCX) [file pone.0260633.s005.docx]

**S1 Table. IL-1β, TNF-α, IL-8 and ACTB primer sequences.**

| Gene Name | Gene Symbol | Primer Sequences  (Forward Primer- Reverse Primer) |
| --- | --- | --- |
| Interleukin-1beta | IL-1β | Forward: 5’-AAGATGCTGGTTCCCTGCC-3’  Reverse: 5’-GCGTGCAGTTCAGTGATCGTAC-3’ |
| Tumor necrosis factor-alpha | TNF-α | Forward: 5’-GGCTTTCGGAACTCACTGGA-3’  Reverse: 5’-CCCGTAGGGCGATTACAGTC-3’ |
| Interleukin-8 | IL-8 | Forward: 5’-CCGGAAGGAACCATCTCACT-3’  Reverse: 5’-ACTTCTCCACAACCCTCTGC-3’ |
| Actin beta | ACTB | Forward: 5’-TCACCCACACTGTGCCCATCTACGA-3’  Reverse: 5’-CAGCGGAACCGCTCATTGCCAATGG-3’ |
