# Supplementary material for: Neuroprotective effects of some epigenetic modifying drugs’ on Chlamydia pneumoniae-induced neuroinflammation: A novel model
Source: PLoS One. 2021 Nov 30;16(11):e0260633. doi: 10.1371/journal.pone.0260633 (PMC8631675; doi:10.1371/journal.pone.0260633)
Supplement: S2 Table — (DOCX) [file pone.0260633.s006.docx]

**S2 Table. RT-PCR cycling conditions.**

| **Step** | **Heat** | **Duration** | **Cycle** |
| --- | --- | --- | --- |
| Incubation | 95° C | 10 min | 1 |
| Amplification | 95° C  60° C  72° C | 10 s  30 s  1 s | 45 |
| Cooling | 40° C | 30 s | 1 |
